# Challenges in implementing an exercise intervention within residential psychiatric care: A mixed methods study

**DOI:** 10.1016/j.mhpa.2017.04.004

**Published:** 2017-03

**Authors:** Joseph Firth, Rebekah Carney, Michelle Pownall, Paul French, Rebecca Elliott, Jack Cotter, Alison R. Yung

**Affiliations:** a Division of Psychology and Mental Health, University of Manchester, UK; b Five Boroughs Partnership NHS Foundation Trust, Greater Manchester, UK; c Greater Manchester West NHS Mental Health Foundation Trust, UK; d Department of Psychological Sciences, The University of Liverpool, UK; e Division of Neuroscience and Experimental Psychology, University of Manchester, UK

**Keywords:** Psychosis, Schizophrenia, Physical health, Physical activity, Bipolar

## Abstract

Physical exercise is increasingly recognized as an important component of psychiatric care, although the feasibility of implementing exercise in residential care settings is not well understood. We evaluated the feasibility of a 10-week intervention of weekly fitness classes (delivered by a personal trainer) and other exercise activities using a mixed-methods approach. This was offered to across four residential care services, to all 51 residents who had severe mental illness (SMI). Of these, 27.5% consented to the exercise intervention. Participants averaged 87.6 min of moderate-to-vigorous exercise per-week, although fitness classes were poorly attended, and 35.7% dropped-out over 10 weeks. Of those who completed the intervention, increased physical activity was associated with significantly reduced negative symptoms. In conclusion, implementing exercise interventions in residential psychiatric care is challenging; given that supervised exercise classes may not be appealing to many residents, while unsupervised exercise is poorly adhered to. Future interventions should consider that better tailored exercise programs are required to adequately confront motivational issues, and to account for participant preference in order to increase engagement.

## Highlights

• This is the first mixed-methods study of exercise in residential psychiatric care.
• The majority of psychiatric patients did not engage with on-site fitness classes.
• Significant reductions in negative symptoms were observed in those who did exercise.
• Symptomatic reductions were significantly correlated with increased physical activity.
• Future interventions may benefit from personalized approaches to exercise support.

## 1 Introduction

Severe mental illnesses (SMI), such as schizophrenia, are associated with a reduced life expectancy of around 15–20 years, which is largely attributed to physical health conditions, such as cardiovascular disease (Laursen, Munk-Olsen, & Vestergaard, 2012). Along with metabolic side-effects of antipsychotic medication, people with SMI also have high rates of smoking, poor diet, and reduced physical activity (Osborn et al., 2007, Stubbs et al., 2016, Vancampfort et al., 2016a). All of these factors are potentially modifiable. For example, small increases in cardiorespiratory fitness can substantially reduce cardiometabolic risk and associated mortality (Kodama et al., 2009). Moderate-to-vigorous exercise can improve mental health through reducing positive and negative symptoms (Firth, Cotter, Elliott, French, & Yung, 2015) and improving cognition (Mrcpuk & Mrcpuk, 2017).

Despite increasing evidence that physical activity improves physical and mental health, the challenge is engaging people with SMI in regular exercise (Vancampfort & Faulkner, 2014). Adherence is often poor and there is high attrition in clinical trials of exercise interventions for SMI (Vancampfort, Rosenbaum, et al., 2016), diminishing their clinical applicability. Optimal methods for delivering exercise in mental health settings have yet to be established Faulker & Taylor, 2009.

This study aimed to evaluate the feasibility of providing on-site fitness classes and professional exercise support within residential care centers for people with SMI. We used a mixed-methods design, combining quantitative and qualitative methods.

## 2 Methods

### 2.1 Setting and participants

The study was approved by the North West Research Ethics Committee on 02/06/2015 (REC# 13/NW/0784). Participants were recruited from four residential psychiatric care centers managed by ‘Alternative Futures Group’ (AFG) around Greater Manchester (UK). Inclusion criteria for the study were: (i) aged 18–65, (ii) receiving treatment for an SMI within an AFG center. Exclusion criteria were the inability to provide informed consent, pregnancy, insufficient English language to complete assessments, or physical health issues which contraindicated exercise (including poorly controlled asthma, diagnosed heart conditions and untreated hypertension) as determined by the referring clinician and general practitioner if required.

### 2.2 Intervention

Participants were asked to engage in 90 min of structured exercise per week for 10 weeks, as this amount has previously been found to improve mental health in SMI (Firth et al., 2015). This was achieved over two weekly sessions; once in an ‘AFG Exercise Class’ held on-site at the rehabilitation center, and once in a further weekly activity of their own choosing.

Exercise classes were conducted by a Personal Trainer (UK Level 4 Qualification) who was familiar with mental health, having worked as an SMI support worker prior to this role. Each session consisted of: 10 min warm up, 45 min circuit training, 5 min cool down. Circuits consisted of 8–16 different stations of aerobic/resistance exercises selected to improve cardiovascular fitness, muscular strength and functional capacity. Stations were varied each week, but typically included exercises such as press-ups, star jumps, lunges, shoulder press, kettle-bell training and abdominal crunches. Each exercise was performed for 1-min, followed by 30 s of rest. The number of repetitions expected of each participant at each of the training stations of the exercise circuit, and the amount of weight used, was tailored to match their physical capabilities; while aiming to maintain a vigorous intensity of exercise throughout. Attendance was encouraged through posters around the AFG centers advertising the weekly classes, entitled ‘Alternative Futures Exercise Classes’ and displaying the day and time of the sessions, along with graphical cartoon images of people exercising. Furthermore, all residents were made personally aware of the classes at the beginning of the intervention, through notification from their clinicians in the form of an invitation letter providing information about the study (see Supplement 1).

For their second weekly session, participants were supported to engage in an activity of their choice by their care team. No behavioral change techniques were systematically applied within this support (Michie et al., 2011), although carers were involved in helping participants to select a preferred activity (such as community walks, on-site gardening, gym training with other service users or cycling with support staff), and providing verbal reminders/encouragement to the participants to attend their scheduled sessions on the given day. Participants were also offered an ‘exercise log book’ to record their weekly exercise. Where participants were not able or interested in using this, AFG staff also recorded exercise attendance for the participant.

### 2.3 Outcome assessment

The primary outcome of this study was feasibility; including recruitment rate, retention, and amount of exercise achieved. Recruitment rate was calculated as the number of service users consented over the 4-week recruitment period, divided by total number of service users at the four centers. Retention was the percentage of enrolled participants who completed follow-up assessments. Amount of exercise was measured using logbooks and the ‘IPAQ’ International Physical Activity Questionnaire (IPAQ Committee, 2005), administered at baseline and follow-up. The IPAQ was used to calculate total change in ‘metabolic equivalent task minutes’ (METs). This score represents the total time and intensity of physical activity engaged in during the last week, indexed as multiples of the resting metabolic rate. For example, walking is scored as 3.3 METs per-minute (IPAQ Committee, 2005) and thus 10 min per-day would yield a weekly score of 231 METs, whereas 1 h of circuit training (which scores 8 METs per-minute) would be 480 METs.

### 2.4 Participant centered outcomes

Participants completed a 10-item ‘tick box’ survey assessing their desired outcomes of exercise when entering the study. Additionally, motivation towards exercise was examined in the context of Self Determination Theory using the ‘Behavioural Regulation in Exercise Questionnaire 2’ (BREQ-2) (Markland & Tobin, 2004) at the end of the study. In line with a previous validation study of the BREQ-2 in SMI populations (Vancampfort et al., 2015), the items for ‘identified regulation’ (personal identification with the reasons for being physically active) were combined with items for ‘intrinsic regulation’ (enjoyment of exercise for its own sake), to calculate a single ‘autonomous motivation’ score which has previously been shown to predict physical activity levels in SMI (Vancampfort et al., 2015). A qualitative sub-study was also used to capture detailed information on participants' experiences of undertaking exercise.

### 2.5 Secondary outcomes

Validated measures of psychiatric symptoms, functioning, and physical health were administered pre- and post-intervention to investigate which areas could feasibly show significant improvement from 10 weeks of exercise. The specific measures used were selected to match those of a recent study of an exercise intervention in community psychiatric care (Firth, Carney, Elliott et al., 2016). The principal change outcome was psychiatric symptoms. This was assessed using the Positive and Negative Syndrome Scale (PANSS) (Kay, Flszbein, & Opfer, 1987). Psychosocial functioning was measured using the Social and Occupational Functioning Assessment Scale (SOFAS) (Morosini, Magliano, Brambilla, Ugolini, & Pioli, 2000). Neurocognitive functioning was assessed on a computerized system, using various tasks available on the default PEBL battery (Mueller & Piper, 2014). Tasks were selected to test multiple cognitive domains including; processing speed (Trail Making Test-A and Letter-Digit Coding), short term verbal memory (12-word immediate free recall), executive functioning (Trail Making Test-B), and visuo-spatial memory (Corsi Tapping Blocks) and motor speed (Finger Tapping). Bodyweight was measured using digital scales, with participants fully clothed although with no footwear.

### 2.6 Procedure

Recruitment took place over a 4-week period in June/July 2015 prior to commencing the intervention. Recruitment was conducted by the AFG nurses and support staff at the four sites, who advertised the exercise classes using flyers and posters and personally informed service users of the study. No monetary incentive was used. Any service users who expressed an interest were provided with a study information sheet. They then met with the research assistant to provide written informed consent.

Semi-structured interviews were conducted within 2 weeks of completing the intervention on a one-to-one basis. All those initially recruited to the main study were asked to take part in the qualitative sub-section, regardless of their exercise participation. The interview topic guide explored service users’ opinion in: *(1) Engagement in exercise* and *(2) Effects of exercise.*

### 2.7 Data analysis

Statistical analyses were conducted in SPSS 20 (SPSS, 2011). Shapiro–Wilk tests and normal probability plots were used to check for normality. A ‘full analysis set’ approach was applied throughout to include all available data for every outcome (Gupta, 2011). Feasibility measures were summarized with sample means and percentages. Changes in physical and mental health outcomes pre- and post-intervention were analyzed using within-subjects t-tests, with significance level of α = 0.05. Effect sizes were calculated as Cohen's d and 95% confidence interventions (CI) were displayed. Bonferroni-corrections for multiple testing were not applied to secondary outcomes due to the exploratory nature of these analyses.

For the qualitative sub-study, the five stages of thematic analysis (Braun & Clarke, 2006) were applied to determine the key themes in service users’ dialogue. To reduce bias, the quality of the emergent themes and sub-themes were reviewed by three authors. Supporting quotes are presented for each theme in Table 1.

**Table 1:** Supporting examples for themes in qualitative data.

Themes	Example quotes
**1. Engagement in Exercise**	
1a. Exercising to improve physical health	P001: “It's worth it if it's going to help me lose weight … I would like to get fitter and feel more healthy and that”. P004:: “I knew that I should to do something; my belly, with my posture, with my health … Because exercise, physical activity, is good for health, so I know that I should to do something. I thought about joining gym, but it's a little bit, it's, you know … it was very good that the exercise was in the place [the AFG center]”
	P006: “… even though I do want to lose weight, a lot of weight, I would say just keeping fit …. “Yeah I think if you start keeping fit, your weight will come with it and eating right.”
1b. Motivation as a primary barrier	P004: “Some people, don't bother about it. So, maybe it was that time that people didn't want to join” …. ”Maybe they gave up, simply. Maybe they didn't realise that it’s hard maybe. Maybe they was too lazy. They didn't bother about it. They maybe gave up themselves.” P005: “It's other people's motivation as well, some people lift you up and some people drag you down with their motivation, and I think there has been a lot of that going on in here …. some people want to and some people don't … It affects your motivation and things, cause some people have low morale, so that doesn't help.” P006: “I must admit, its something I've got to push myself to do, once I'm there and I'm doing it I'm alright but it's just getting there” … “some days I want to just get up and go, most days at the moment, but I just wish sometimes I just had a little more go in me”
1c. Physical goals prompting vigorous exercise	P001: “I know there's one lad, he's saying it's not doing nothing for you, you know. Like, you know, doing all this work, it's not doing nothing, or it's just, I don't know. It's doing daft circuits but it's not about that” …. “I was doing it for myself as well and prove to them that I can do it, you know what I mean. I know the benefits is for yourself. I know it is gonna work” P004: “Everyone has own motivation. Everybody has own desire to, I think should be to reach what motivates them to do something and try to play on this, on that, find it and try to convince them that exercise are good for them and find reason why they should to do it.”
	P006: “Yeah I think its laziness a lot of it, and it can be quite difficult starting but like I keep saying if you want to get to your goals, you've got to just do it ….you want to get to your goals you've just got to go, simple as”
1d. Professional and personal support as the key facilitator	P001: “I don't do enough exercise because I feel like I need someone to like, get me going and give me confidence and get me into the routine. Like, I couldn't go and do it myself, you know do the exercises and all that or sit ups, press and doing weights. It's nice when you got someone with you to like a one to one or to motivate you. P002: “Without a trainer I never would have done it”. “He knows what each exercise will do to your body …. So he's making sure that we exercise to our optimum … he's very good at it and it pushes you which is good as well because when you want to stop and somebody pushes you, you get more out of it.” P004:: It would be very good. If I would be in here and if, for example, someone who knows about exercise would take me to the gym as a coach and show me what to do exercise, what to do to achieve this, this, this. How to make exercise, what exercise, which exercise. I would be very pressured, it would be good for me, for me.
**2. Effects of exercise**	
2a. Replacing negative emotions with positive feelings.	P001:“Once you get on with it, just do a little bit every now and then, every time you felt stressed or like a bit pissed off, do a bit of exercise and then it will calm you down” … “it's just like garden, it's therapeutic isn't it, doing the rest of the garden, you don't realise after you've done it you feel like laid back, and that's the same with doing exercise and you know, gym stuff, you feel open and relaxed.” P005: “It reduces the erm, the, I notice a lot of stress around. It reduces stress.” … “I sleep better of an evening, of a night time. Just feel relaxed. P006: “Sometimes I can just be in a terrible mood at times and I get to the gym and have a blow out like and I feel great sort of thing after it”

2b. Expelling negative states through exercise	P001: But yeah, you can feel very bad before you go and then you come home, you like, you could run for a mile, you could do more, you know what I mean, you just feel good.. P006 “Just doing cardio and things like that, just gets it out of your system” … “Sometimes when I get stressed out and go to the gym, it gets rid of all the stress, so yeah, the mood will get better as well, it does with me anyway” P003: “Well you could use it as a bit of a therapy couldn't you … A bit of, mind off things and just focussing on what you're up to on the treadmill, I think it could help.”

2c. Exercise stimulating positive neurobiological responses	P002:: Well if you're exercising, like, circuit training, releasing endorphins … But when you've stopped exercising it doesn't release them as much on a daily basis. P003: “You feel all the endorphins coming in your brain when you work out …. you know. The chemicals in your brain afterwards, the endorphins, you feel better.” P005: It's quite good, yeah. It's quite good. The serotonin levels releasing and endorphins there so it's quite good, yeah.

## 3 Results

### 3.1 Recruitment and retention

**Fig. 1:**
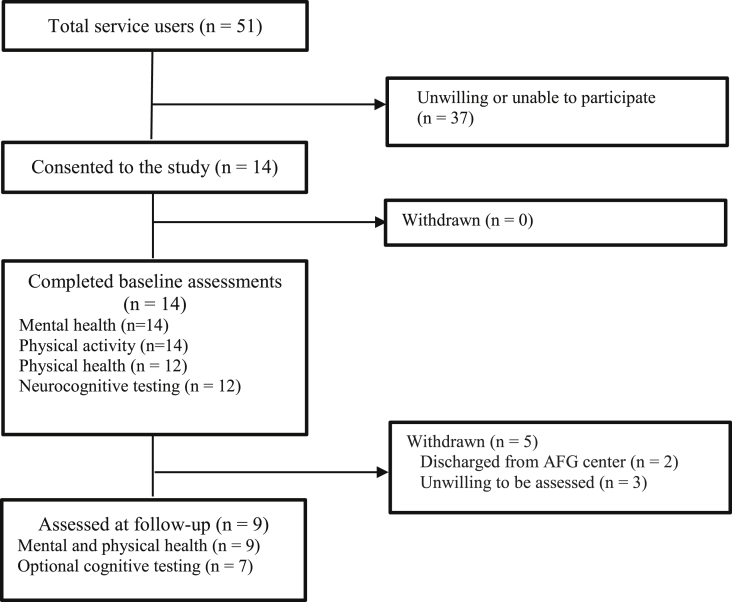
Flow diagram of recruitment and retention.

Recruitment and retention are displayed in Fig. 1. Recruitment rate was 27.5% of the entire target sample, as only 14 of all 51 people residing across the four AFG centers consented and completed baseline assessments. Retention over 10-weeks was 64.3%; with 5/14 participants dropping-out. Characteristics of completers and withdrawals are compared in Table 2, showing that those who dropped out had higher positive symptoms.

**Table 2:** Baseline characteristics of participants.

	Total Sample (n = 14)	Completers (n = 9)	Withdrawals (n = 5)
Gender			
Male; n (%)	10 (71)	12 (86)	3 (60)
Female; n (%)	4 (29)	2 (14)	2 (40)
Age, years; mean (s.d.)	38.5 (9.8)	38.3 (8.6)	38.8 (12.8)
Months in service; mean (s.d.)	18.9 (13.6)	20 (14.2)	17 (13.7)
Duration of SMI; mean (s.d.)	13.4 (7.9)	15.3 (7)	9.0 (9.6)
Physical Health; mean (s.d.)			
Bodyweight, kg	103.7 (24)	106.8 (24)	94.3 (28)
Physical activity, METs	942 (773)	986 (909)	862 (529)
Mental Health; mean (s.d.)			
PANSS total symptoms	94.5 (20.1)	89.1 (19.7)	104.2 (18.9)
PANSS positive symptoms^a^	22.2 (6.5)	19.3 (4.8)	27.4 (6.2)
PANSS negative symptoms	24.9 (5.9)	24.6 (6.6)	25.4 (4.9)
PANSS general symptoms	47.4 (5.9)	45.2 (11)	51.4 (10)
SOFAS score symptoms	46.3 (5.7)	46.0 (6.5)	46.8 (4.6)

a Significant difference between completers and withdrawals (p < 0.05) PANSS, Positive and Negative Syndrome Scale; SOFAS, Social and Occupational Functioning Assessment Scale.

### 3.2 Exercise achieved

In the 10-week assessments, total weekly activity had non-significantly increased by 43% from baseline (*d* = 0.47, 95% C.I. = −543 to +1383 METs, p = 0.344). Participants’ autonomous regulation towards exercise held strong, positive correlations with their levels of physical activity at the 10-week follow-up (r_s_ = 0.745, p = 0.021). Exercise logbooks were completed by 8 participants, who engaged in 87.4 min of moderate-to-vigorous exercise per-week. Only 42% of this exercise was achieved through AFG exercise classes, with the remaining amounts being accounted for by gym sessions, dance classes and/or cycling. Participants achieved 141 min of low-intensity activity per-week, mostly through walking.

### 3.3 Participant centered outcomes

The most common incentives for exercise were physical health, with ‘Increase fitness/energy’ selected by 92.9%, closely followed by ‘Lose weight’ and ‘Increase sporting ability/strength’ (both 78.6%). The most common psychosocial incentives were ‘Having fun’ (78.6%) and ‘Taking your mind off things’ (64.3%).

All participants were invited to qualitative interviews, of which 6 agreed (all who had completed the intervention). The key emergent themes were in relation to (i) physical health as the primary incentive for exercise, (ii) low motivation been the primary barrier, (iii) importance of professional support, and (iv) acute benefits of exercise on affective state. The emergent themes and supporting quotes are detailed in Table 2.

#### 3.3.1 Quantitative changes following exercise

Total symptom scores reduced non-significantly, by 12.2% or −7.2 PANSS points (*d*=0.295, 95% C.I. = -15.3 to 0.88 points, p = 0.074). PANSS negative symptoms showed greatest improvement, reducing by 21% (*d*=0.55, 95% C.I. = -7.35 to 0.02 points, p = 0.051). Positive symptoms and general symptoms reduced by 11.7% (*d* = 0.297, 95% C.I. = -4.4 to 1.5 points, p = 0.252) and 11.8% (*d* = 204, 95% C.I. = -8.2 to 1.3 points, p = 0.133), respectively. Exploratory post-hoc analyses found a significant association between increases in physical activity (METs) and decrease in psychiatric symptoms (PANSS total) (r_s_ = 0.77, p = 0.015). Increases in physical activity were significantly correlated with decreases in negative symptoms (r_s_ = 0.681, p = 0.044) and general symptoms (r_s_ = 0.75, p = 0.02). The neurocognitive task battery was completed by fewer participants at follow-up (n = 7) and there were no significant changes in any cognitive domain. The 6.5% improvement in socio-occupational functioning was also non-significant (*d* = 0.478, 95% C.I. = -3.3 to 9.3 SOFAS points, p = 0.302). Although participants lost an average of 0.33 kg (kg), there were no significant changes in bodyweight (*d*=0.014, 95% C.I. = -1.8–2.47 kg, p = 0.729).

## 4 Discussion

### 4.1 Feasibility of exercise interventions for SMI

This study combined quantitative and qualitative data sources to evaluate the implementation of weekly exercise classes in residential care settings. The primary finding was only a minority of patients with SMI were interested in participating in the exercise intervention; with only 27.5% of service users joining the study. Unfortunately, it was not possible to gather data from non-participating service users, although interviews with those who consented indicated that the main reason for the low participation rate was a lack of motivation towards exercise. This suggests that future interventions should employ more than informational/promotional materials when attempting to engage this population in regular exercise. For instance, previous study by Beebe et al. (2011) showed that a motivational intervention aimed at promoting physical activity among people with schizophrenia successfully increased walking among long-term patients, without actually delivering walking sessions, but instead focusing addressing barriers, teaching about benefits of exercise and cueing walking behaviour.

Among those who joined our intervention, the most common reasons for taking up exercise were improving fitness and losing weight, which corresponds with large-scale survey data from this population (Firth, Rosenbaum et al., 2016). While some participants' physical health goals continuously motivated them, those who doubted the effectiveness of the exercise classes for reaching their goals had no incentive to participate. Previous studies have found that people with SMI with ‘autonomous motivation’ towards exercise (i.e. those who identify with the benefits of a healthy lifestyle and feel intrinsically motivated towards it) are more likely to engage in regular physical activity (Vancampfort et al., 2015). Consistent with this, we found that autonomous motivation scores were significantly correlated with exercise participation. These findings indicate that supporting people with SMI to achieve their personal goals through exercise may increase engagement. Future interventions could use motivational interviewing to direct people towards types of activities which match their preferences and desired outcomes, and provide a more varied range of supervised exercise activities (rather than just circuit training).

### 4.2 Changes associated with exercise

Despite low adherence overall, those who engaged with the intervention demonstrated some notable benefits after just 10 weeks., The largest improvements were in negative symptoms. These symptoms are strongly associated with long-term functional impairment, and tend not respond to antipsychotics (Kirkpatrick, Fenton, Carpenter, & Marder, 2006). These clinically meaningful improvements corresponded with the emergent themes of qualitative interviews, which found acute ‘feel good effects’ from moderate-to-vigorous exercise, as found other qualitative studies (Firth, Carney, Jerome et al., 2016). However, there were minimal changes in bodyweight following the intervention.

The age of participants (39 years) and their duration of SMI (15 years) are important factors to consider when interpreting these findings. Previous research in people with long-term SMI has suffered from high rates of attrition and found that benefits only occur for the subset of patients who adhere to the interventions provided (Scheewe et al., 2013, Vancampfort et al., 2016b). This may be due to long-term antipsychotic treatment, and associated sedentary habits, obesity and metabolic complications, all of which act as a barrier to exercise (Vancampfort et al., 2012). Nonetheless, broader lifestyle interventions which have proven effective for reducing bodyweight, even in long-term obese patients. For example, Daumit et al. (2011) combined group exercise sessions with weight-management counselling; incorporating social cognitive theory and behavioral self-management in a manner which had previously proven effective in non-psychiatric populations, but was adapted for psychiatric inpatients in this study. This attracted higher rates of participation than our intervention, with 64% of eligible residents joining the study, high rates of adherence over 6 months, and significant reductions in bodyweight and waist circumference.

### 4.3 Limitations

Although the scale of this evaluation project was reasonably large (four centers, 51 patients), only a small proportion consented to take part in exercise, reducing our ability to generalize from these findings. Along with continuing to study the benefits in patients who volunteer for exercise trials, future research should explore new ways to reach and engage the majority of people with SMI, who may typically opt-out of exercise or regular physical activity (Soundy et al., 2013, Stubbs et al., 2016). Developing interventions which draw on and support people's autonomous motivation may be effective for increasing motivation towards exercise in this patient group (Vancampfort et al., 2015). Additionally, focusing on motivational aspects of physical activity engagement, rather than just providing exercise sessions, may be more important for increasing physical activity in long-term patients (Beebe et al., 2011).

Another limitation of this study is that the only form of supervised exercise offered to participants was circuit training classes. Therefore, the lack of participation could be due to some service users having a general interest in exercise, but are averse to this specific format. Thus, more targeted and personalized approaches to exercise coaching (rather than offering general classes) may benefit greater numbers of service users; as has been observed in other studies which tailor exercise interventions towards participant preference (Bartels et al., 2014, Firth et al., 2016a). The role of qualified exercise professionals in mental healthcare services could be extended beyond providing exercise classes to also include facilitating engagement in exercise activities available in service users local community; especially for those who feel unable to attend these activities alone (Bartels et al., 2014).

## Funding

This study was funded by Greater Manchester West NHS Mental Health Foundation Trust. Corresponding author JF is funded by an MRC Doctoral Training Grant (P117413F07).

## Competing interests

All authors declare that they have no competing interests.
