# Supplementary material for: Challenges in implementing an exercise intervention within residential psychiatric care: A mixed methods study
Source: Ment Health Phys Act. 2017 Mar;12:141–6. doi: 10.1016/j.mhpa.2017.04.004 (PMC5455809; doi:10.1016/j.mhpa.2017.04.004)
Supplement: Supplementary [file mmc1.pdf]

Participant Information Sheet

You have been given this information sheet because you are being invited to take part in a research project. It is up to you to decide whether or not you want to take part. Before you decide, it is important for you to understand why the research is being done and what it will involve. Please take as much time as you need to read and understand this information. If there is anything you don't understand you can speak to. Feel free to ask a member of the iBeep team if anything is not clear, or if you would like more information. Contact details can be found at the end of this information sheet.

What is the purpose of the study?

Some people who experience psychological difficulties sometimes find it hard to feel motivated, and may lack energy and feel low in mood. We believe that physical exercise may help with problems such as low mood, anxiety, irritability, and improve physical health. This project is being done to see if increasing physical activity can reduce some of these difficulties and promote physical health.

Why have I been invited to take part?

We are inviting people who receive support from Alternative Futures Group to take part. If you are interested in taking part we will contact you to ask some questions about your experiences, to help us decide whether this project is right for you.

Do I have to take part?

No, it is up to you to decide whether or not you want to take part. You can change your mind at any time without having to give a reason. If you decide not to take part, or to stop taking part, this will not affect the standard of care you receive.

What will be involved in the exercise plan?

The intervention is a physical activity programme. We want to examine the effects of exercise on symptoms such as depressed mood, anxiety, social functioning, memory, and physical health. If you choose to take part, our Personal Trainer will be happy to guide you in exercise classes, and will also be happy to advise you on any other physical activity you may want to do. Your exercise plan can be flexible to change, and the activities, times and locations will be based on your personal preference.

What will happen if I decide to take part?

If you inform any member of AFG staff that you would like to participate in the study, your contact details will be passed on to a member of the study team. You will then receive a phone call from an Research Assistant to arrange to see you at a convenient place to let you sign up to the study and complete the assessments.
